# Impact of Combined Interventions and Early Home Care Activation on 30-Day Hospital Readmissions: A Retrospective Observational Study

**DOI:** 10.3390/medicina62030602

**Published:** 2026-03-23

**Authors:** Gianluca Azzellino, Patrizia Vagnarelli, Ernesto Aitella, Francesca Cerratti, Luca Mengoli, Lia Ginaldi, Massimo De Martinis

**Affiliations:** 1 Department of Life, Health and Environmental Sciences, University of L’Aquila, 67100 L’Aquila, Italy; 2 U.O.C. Adriatic District Area, Azienda Unità Sanitaria Locale 04 Teramo, 64100 Teramo, Italy; 3 Allergy and Clinical Immunology Unit, Center for the Diagnosis and Treatment of Osteoporosis, Azienda Unità Sanitaria Locale 04 Teramo, 64100 Teramo, Italy; 4 Department of Medicine and Science of Aging, “G. D’Annunzio” University of Chieti, 66100 Chieti, Italy; 5 Long-Term Care Unit, “Maria SS. dello Splendore” Hospital, AUSL 04 Teramo, 64021 Giulianova, Italy; 6 UniCamillus-Saint Camillus International University of Health Sciences, 00131 Rome, Italy

**Keywords:** hospital readmission, transitional care, nurse case management, integrated home care, hospital-to-community transition, frail older adults, discharge planning, continuity of care

## Abstract

*Background and Objectives*: Thirty-day hospital readmissions are a major clinical and economic challenge, particularly among frail older patients. Integrated protected discharge models, coordinated by nurse case managers and including multidimensional hospital-to-community interventions, may improve continuity of care and reduce inappropriate readmissions. *Materials and Methods*: We conducted a retrospective observational study on 200 consecutive patients aged ≥65 years, discharged between January and December 2024 from a public hospital in Italy. Frailty was assessed using BRASS scores (≥11), ADL, and IADL. The primary outcome was 30-day hospital readmission. Logistic regression models evaluated the impact of individual interventions (Model 1) and combined interventions (Model 2), defined as the simultaneous presence of four components: nurse case manager involvement, telephone follow-up, activation of home care (ADI) within 24 h, and social worker support. *Results*: Overall, 65 patients (32.5%) were readmitted within 30 days. In the multivariate analysis, nurse case manager involvement was associated with lower odds of readmission (OR = 0.023; 95% CI 0.008–0.064; *p* < 0.001). Early ADI activation was not associated with readmission in the bivariate analysis (*p* = 0.195) but showed higher odds of readmission in the multivariable model (OR = 3.475; 95% CI 1.384–8.725; *p* = 0.008). The combined interventions variable was significantly associated with readmission in Model 2. Patients who did not receive combined interventions had higher odds of 30-day hospital readmission compared with those who did (OR = 26.1; 95% CI 10.1–67.5; *p* < 0.001). *Conclusions*: An integrated protected discharge model coordinated by a nurse case manager and including combined interventions was associated with lower odds of 30-day hospital readmission among frail older patients. These findings suggest that the potential benefit of transitional care may lie not in isolated interventions but in the multidimensional integration and coordination of clinical, functional, and social support strategies, highlighting the central role of nurse case managers in transitional care pathways.

## 1. Introduction

Thirty-day hospital readmissions represent a major clinical and economic challenge for healthcare systems, particularly among older adults with chronic conditions and functional dependency. Unplanned readmissions are associated with poorer outcomes, increased caregiver burden, and higher costs, and are widely considered an indicator of quality and continuity of care across settings [1,2,3,4]. In frail populations, the risk of early readmission is multifactorial, reflecting not only clinical severity but also functional impairment, adverse events during hospitalization, and social vulnerability [5,6,7]. These factors highlight the need for structured, cross-setting models that support safe transitions from hospital to community care. To address preventable readmissions, a range of transitional care and discharge planning strategies has been developed, including comprehensive discharge planning, nurse-led case management, early post-discharge contact, and telephone follow-up [2,8,9,10,11,12,13]. Evidence suggests that nurse-led interventions can improve continuity and reduce readmissions when they include multidimensional assessment, patient/caregiver education, and timely follow-up [2,11,12,14,15,16]. Telephone follow-up has been repeatedly described as a practical component of transitional care, with potential benefits in early identification of deterioration, reinforcement of discharge instructions, and support for caregivers [17,18,19]. In parallel, the involvement of social services may address social determinants of readmission—such as limited family support, disadvantaged socioeconomic conditions, and reduced access to community resources—thereby strengthening discharge readiness and linkage to territorial services [6,7,20,21]. Nonetheless, the effectiveness of single interventions remains heterogeneous across settings and populations, and the literature continues to report mixed results, suggesting that success may depend on how interventions are integrated and operationalized within local care pathways [8,9,13,22,23,24]. Within this context, risk stratification tools and functional measures are commonly used to identify patients who may benefit most from structured discharge planning. The BRASS index has long been used to screen patients requiring personalized discharge planning [25], and functional dependency measures such as ADL and IADL have been associated with adverse post-discharge outcomes in older adults [26,27,28]. However, risk identification alone is insufficient if not paired with coordinated transitional care actions and timely activation of community services [9,13,16]. In Italy, this need aligns with the ongoing reorganization of territorial care and integrated hospital–community models, where transitional care and proactive follow-up have become core priorities [29,30,31]. Moreover, recent nursing perspectives emphasize that the combination of predictive assessment tools with structured follow-up and telemedicine may further enhance early risk identification, proactive discharge planning, and post-discharge monitoring—particularly for chronic and frail patients transitioning across care settings [32,33]. Despite this evolving evidence base, an important gap remains: it is still unclear whether a combined, multidimensional package of transitional care actions—rather than isolated components—provides the greatest protection against 30-day readmissions in frail older patients, especially in real-world routine practice. Few observational studies have evaluated the “synergistic” effect of coordinated interventions that jointly address clinical, functional, and social dimensions of discharge. Therefore, this retrospective observational study aimed to evaluate the impact of an integrated protected discharge model, coordinated by a nurse case manager and targeting patients aged ≥65 years at high risk of readmission, in which nurse case management, nurse-led telephone follow-up, early activation of home care services (ADI) within 24 h, and social worker support are implemented as a coordinated set of interventions designed to reduce 30-day hospital readmissions.

## 2. Materials and Methods

### 2.1. Study Design, Setting, and Population

A retrospective observational study was conducted among patients discharged between January and December 2024 from a public hospital in the Abruzzo region (Italy), with the aim of examining the association between a structured protected discharge model, coordinated by a nurse case manager, and the risk of 30-day hospital readmission in frail older patients.

The protected discharge model consisted of four coordinated components delivered during the hospital-to-community transition: (1) nurse case manager involvement, (2) social worker support, (3) activation of Integrated Home Care services (ADI) within 24 h after discharge, and (4) post-discharge nurse-led telephone follow-up.

### 2.2. Intervention Components

#### 2.2.1. Nurse Case Manager (CM)

The nurse case manager was a hospital-based nurse responsible for coordinating discharge planning for patients identified as high risk (BRASS ≥ 11). A multidimensional assessment was performed within 48–72 h prior to discharge and included evaluation of functional status (ADL, IADL), medication reconciliation, caregiver availability, and home care needs. The CM coordinated communication between hospital physicians, ward nurses, home care services (ADI), and social services when required. Discharge planning activities and referrals were documented in the institutional electronic medical record.

#### 2.2.2. Social Worker Support

Social worker involvement was activated for patients presenting social vulnerability, limited caregiver support, or complex discharge needs, as identified by the nurse case manager or ward team. The social worker conducted a structured socio-environmental assessment addressing housing conditions, family support, socioeconomic constraints, and access to community resources. When indicated, referrals to municipal services, social assistance programs, or long-term care facilities were facilitated. Social work interventions were recorded in the institutional information system.

#### 2.2.3. Activation of Integrated Home Care (ADI)

Activation of ADI was initiated prior to discharge by the nurse case manager in coordination with the multidisciplinary hospital team. Early activation was defined as the first home care contact occurring within 24 h after discharge. ADI services included nursing assessment, clinical monitoring, medication management support, and coordination with general practitioners and community services. Although nursing care represented the primary component documented in the institutional databases, ADI is intrinsically a multidisciplinary service that may also involve physicians, physiotherapists, and other healthcare professionals according to patient needs. Timing of activation was verified through the home care registry.

#### 2.2.4. Post-Discharge Telephone Follow-Up

A structured telephone follow-up was conducted by the nurse case manager within 48–72 h after discharge. The call aimed to assess clinical stability, adherence to prescribed therapy, understanding of discharge instructions, and caregiver needs. If clinical or organizational issues were identified, appropriate referrals to ADI services, general practitioners, or hospital services were arranged. Telephone contacts were documented in the electronic medical record.

The protected discharge pathway has been integrated into routine hospital discharge procedures since 2018 and is activated following systematic BRASS screening during hospitalization. Eligible patients were referred to the nurse case manager for coordination of the discharge plan. Each intervention component could be delivered individually according to patient needs and clinical judgment. For analytical purposes, a dichotomous variable (“combined interventions”) was subsequently constructed to identify patients who received all four components. The operational workflow included: patient identification through risk screening, multidimensional assessment, coordination with territorial services, early activation of home care, and structured post-discharge follow-up.

### 2.3. Sample and Inclusion/Exclusion Criteria

Patients aged ≥65 years with chronic conditions and frailty were included. In line with the literature, a BRASS score ≥ 11 was used to identify individuals at high risk of hospital readmission [25]. Frailty was further assessed using functional dependency scales: ADL [27] and IADL [28]. Patients transferred to facilities other than ADI or enrolled in palliative care for terminal conditions were excluded. The total sample consisted of 200 patients, corresponding to all consecutive eligible cases during the observation period. No formal sample size calculation was performed, as the study had a retrospective design. This aspect, together with the number of variables included in the multivariate model, exposes the analysis to the risk of overfitting, which is addressed in Section 4.1.

#### 2.3.1. Data Collection

For each patient, the following information was retrospectively extracted from electronic databases: demographic characteristics (age and sex), functional scores (BRASS, ADL, and IADL), discharge ward, timing and modality of home care (ADI) activation, involvement of the nurse case manager and the social worker, and post-discharge telephone follow-up. The discharge ward (e.g., Internal Medicine, Orthopedics, Cardiology, Surgery, Long-term Care) was used as a proxy indicator of the primary clinical context and case mix of the index hospitalization and was described in the dataset; however, it was not included in the final regression models.

The primary outcome was hospital readmission within 30 days of discharge.

Candidate independent variables included age, sex, functional scores (BRASS, ADL, and IADL), discharge ward, timing of ADI activation, and involvement of different professionals (nurse case manager, social worker, and post-discharge telephone follow-up).

To assess the integrated effect of the different actions included in the protected discharge model, a dichotomous variable named “combined interventions” was constructed. This variable was coded as 1 (yes) if the patient had received all key components (involvement of the nurse case manager, nurse-led telephone follow-up, timely activation of ADI within 24 h, and social worker support) and 0 (no) otherwise. The composite variable was intentionally defined as receipt of all four components to represent full implementation of the protected discharge pathway. Given the limited sample size and the sparse distribution of partial combinations, more granular pattern analyses (e.g., specific combinations or dose–response) were not performed in order to avoid unstable estimates.

Readmissions were verified exclusively through the electronic medical records of the institution and included only hospitalizations occurring in one of the hospitals of the local health authority within 30 days of discharge. Emergency department visits without subsequent hospitalization were not considered readmissions, as the study focused specifically on hospital readmissions requiring inpatient admission, which represent a more robust indicator of care transition failure. The timing of ADI activation was recorded with particular attention to early activation. Missing temporal data accounted for less than 5% of the sample. These patients were retained in the overall dataset but excluded from analyses involving the timing of ADI activation in order to minimize potential bias.

#### 2.3.2. Additional Home-Based Services

Some patients may have received additional home-based services (e.g., physiotherapy, specialist consultations, or privately arranged assistance) outside the hospital-coordinated discharge pathway. These services were not systematically recorded in the institutional electronic databases and therefore could not be included in the statistical adjustment. Consequently, their potential influence on 30-day readmission risk cannot be excluded.

All data were anonymized prior to analysis, in accordance with national regulations and the General Data Protection Regulation [34]. Since the study relied exclusively on pre-existing anonymized data, ethics committee approval and informed consent were not required.

The study was conducted and reported in accordance with the STROBE (Strengthening the Reporting of Observational Studies in Epidemiology) recommendations for observational studies [35]. The completed STROBE checklist is provided as Supplementary Materials.

#### 2.3.3. Statistical Analysis

The Shapiro–Wilk test showed that none of the variables followed a normal distribution. Therefore, medians and interquartile ranges (IQRs) were reported. Categorical variables were described as frequencies and percentages.

**Bivariate analysis:** group comparisons (readmitted vs. non-readmitted patients) were performed using the non-parametric Mann–Whitney U test for continuous variables and the Pearson Chi-square test, or Fisher’s exact test when expected frequencies were <5, for categorical variables.

**Multivariate analysis:** A multivariate logistic regression model was applied to identify independent predictors of 30-day readmission. Given the potential overlap and co-occurrence among intervention components (nurse case manager involvement, social worker support, early ADI activation, and telephone follow-up), two separate models were developed in order to reduce multicollinearity and better estimate their independent and combined effects.

**Model 1** was initially specified to include clinical variables (age, sex, ADL, IADL, and BRASS) and individual intervention components (nurse case manager, ADI within 24 h, social worker involvement, and telephone follow-up). Because of strong collinearity among some intervention components, only nurse case manager involvement and ADI within 24 h were retained in the final model.

**Model 2** included the same clinical variables but replaced individual intervention components with a dichotomous composite variable (“combined interventions”), defined as the simultaneous presence of all four components. This approach was adopted to account for the coordinated nature of the intervention and to mitigate potential collinearity among correlated exposure variables.

Collinearity diagnostics were performed prior to model estimation. Given the high correlation between certain intervention components, the composite variable was used to provide a more parsimonious and interpretable estimate of the overall association of the integrated discharge model.

Effects were expressed as odds ratios (ORs) with 95% confidence intervals (95% CI). The significance level was set at *p* < 0.05. Statistical analyses were performed using IBM SPSS Statistics, version 25 (IBM Corp., Armonk, NY, USA). All statistical analyses were conducted between March and April 2025.

## 3. Results

The study population consisted of 200 patients, of whom 127 were women (63.5%) and 73 were men (36.5%). The median age was 85 years (IQR 79–89). Functional assessment showed a high level of dependency: median ADL was 1 (IQR 0–2), median IADL was 1 (IQR 0–2), and median BRASS score was 20 (IQR 16–22). Baseline characteristics according to 30-day readmission status are presented in Table 1. Hospital readmission within 30 days occurred in 65 patients (32.5%), while 135 patients (67.5%) were not readmitted.

Regarding discharge interventions, nurse case manager involvement, social worker support, and nurse-led telephone follow-up were each recorded in 105 patients (52.5%). Integrated Home Care (ADI) was activated within 24 h of discharge in 164 patients (82.0%). A combined intervention including all these components was implemented in 104 cases (52.0%).

### 3.1. Bivariate Analysis

Bivariate analysis was performed to evaluate the association between patient characteristics and the primary outcome (30-day hospital readmission).

#### 3.1.1. Continuous Variables

Continuous variables were expressed as median and interquartile range (IQR). Comparisons between readmitted and non-readmitted patients were performed using the Mann–Whitney U test.

No significant differences were observed for age (*p* = 0.867), ADL (*p* = 0.149), or IADL (*p* = 0.508). In contrast, BRASS scores were significantly higher among readmitted patients (*p* = 0.006).

#### 3.1.2. Categorical Variables

Categorical variables were compared using Pearson’s Chi-square test or Fisher’s exact test, as appropriate.

No significant association was observed between sex and 30-day readmission (*p* = 0.589). Involvement of the nurse case manager (*p* < 0.001), social worker support (*p* < 0.001), and post-discharge telephone follow-up (*p* < 0.001) were significantly associated with 30-day readmission. Activation of ADI within 24 h was not significantly associated with readmission (*p* = 0.195). The combined interventions variable was significantly associated with 30-day readmission (*p* < 0.001) (Table 2).

### 3.2. Multivariate Analysis

#### 3.2.1. Model 1 (Individual Interventions)

Due to strong collinearity among nurse case manager involvement, social worker support, and telephone follow-up (which largely co-occurred in the same patients), only the nurse case manager variable was retained among these correlated intervention components in Model 1, while ADI within 24 h was kept as a separate independent variable. In routine practice within this organizational pathway, these components were systematically delivered together as part of the same coordinated discharge process. This explains the identical frequencies observed in the dataset and the resulting collinearity between these variables.

The multivariate logistic regression model including clinical variables, nurse case manager involvement, and ADI activation was statistically significant (χ^2^ = 93.58; *p* < 0.001). The model explained 52.1% of the variance in 30-day readmission (Nagelkerke’s R^2^ = 0.521). Classification accuracy was also calculated (83%) but should be interpreted cautiously given the imbalance between outcome groups.

Involvement of the nurse case manager was significantly associated with lower odds of 30-day readmission (OR = 0.023; 95% CI 0.008–0.064; *p* < 0.001). Activation of ADI within 24 h was associated with higher odds of readmission (OR = 3.475; 95% CI 1.384–8.725; *p* = 0.008). Age, sex, ADL, IADL, and BRASS scores were not independently associated with the outcome (Table 3).

#### 3.2.2. Model 2 (Combined Interventions Variable)

The second multivariate model, which included clinical variables and the dichotomous variable “combined interventions,” was statistically significant (χ^2^ = 84.32; *p* < 0.001), with a Nagelkerke’s R^2^ of 0.480. Classification accuracy was also calculated (81.5%) but should be interpreted cautiously given the distribution of the outcome.

The combined interventions variable was independently associated with 30-day readmission (OR = 26.1; 95% CI 10.1–67.5; *p* < 0.001). Patients who did not receive combined interventions had significantly higher odds of readmission compared with those who did. (Table 4).

### 3.3. Predicted Probabilities

To enhance interpretability of the effect size, observed readmission rates were examined according to exposure status. Among patients who received combined interventions, 6 out of 104 (5.8%) were readmitted within 30 days, compared with 59 out of 96 (61.5%) among those who did not receive combined interventions. The absolute difference in readmission probability between groups was 55.7 percentage points.

## 4. Discussion

This study showed that an integrated protected discharge model, coordinated by the nurse case manager and including timely activation of home care (ADI), involvement of the social worker, and post-discharge telephone follow-up, was associated with a lower risk of 30-day hospital readmission in frail older patients. In particular, nurse case manager involvement emerged as a strongly protective predictor, while the adoption of combined interventions was markedly associated with lower readmission rates, highlighting the potential value of a coordinated and multidimensional approach compared with isolated strategies. However, the magnitude of the observed effect sizes should be interpreted with caution, as it may partly reflect the strong imbalance in readmission rates between exposure groups within this specific organizational pathway. The relatively high pseudo-R^2^ values observed in the regression models may partly reflect the strong separation between exposure groups within this single-center organizational pathway rather than a true causal effect. These findings fit within a body of literature characterized by sometimes conflicting evidence. Some studies have reported no significant reduction in readmissions through nurse-led case management programs [36,37], whereas others have demonstrated relevant benefits in terms of continuity of care and reduced hospital length of stay [11,38]. In particular, a systematic review and a randomized clinical trial showed that the success of interventions depends on early post-discharge contact, regular follow-up, patient education, and the involvement of specialized nurses [11,12]. Similarly, a randomized trial showed that structured support programs from hospitalization to return home were associated with better outcomes for older patients [14]. The results of the present study strengthen the evidence in favor of nurse case management, suggesting that the role of the nurse case manager goes beyond technical coordination, extending the effectiveness of interventions through a comprehensive vision that integrates clinical, social, and family aspects. The observed effectiveness is consistent with the literature, which emphasizes that nursing-led transitional care is essential for the success of healthcare reforms and the reduction in inappropriate readmissions [10,39]. A particularly relevant aspect concerns the importance of combined and multidisciplinary interventions. Available evidence shows that multimodal interventions coordinated by interdisciplinary teams are more effective than isolated strategies [2,15,23,31,40]. Moreover, programs involving physicians, nurses, pharmacists, and social workers have been shown to significantly reduce readmission rates [41]. Similar results have been obtained with the “Bridge Model,” in which the involvement of social workers improved the quality of hospital transitions and reduced repeated hospitalizations [21]. More recently, it has been demonstrated that an integrated multidisciplinary approach in patients with heart failure can reduce readmission rates, confirming the relevance of shared care strategies. In the results of this study, isolated activation of ADI was not protective and was instead associated with a higher risk of readmission, probably due to confounding by indication, as patients with greater clinical complexity or higher care needs are more likely to be referred to home care services. In this context, ADI activation may function as a marker of clinical complexity rather than an independent risk factor for readmission. These findings suggest that the apparent association likely reflects patient selection rather than any harmful effect of home care itself. This is consistent with the literature, which has shown that the benefits of home care programs largely depend on integration with other transitional care tools [16,22], and is confirmed by the Italian experience, in which home care models reduced hospitalizations only when structured within a coordinated and multidisciplinary system [42]. The telephone follow-up, delivered within the coordinated discharge pathway, was associated with lower readmission rates. The literature confirms that the active inclusion of caregivers in discharge planning significantly reduces the risk of rehospitalization [43]. Similarly, nurse-led telephone follow-up, which has already proven effective in various settings, was confirmed as a key element in ensuring continuity of care, preventing complications, and detecting early signs of clinical deterioration [17,19]. Regarding assessment tools, our study found that a higher BRASS score was associated with greater risk of readmission in bivariate analysis, but it did not remain an independent predictor in the multivariate analysis. This result is consistent with the literature, where a simplified version of the BRASS index has been proposed to emphasize that clinical-functional assessment alone is insufficient to predict post-discharge outcomes [44]. BRASS remains useful as an initial screening tool, but it is not sufficient on its own: it must be integrated with clinical and social evaluations. Similarly, it has been shown that risk assessment and discharge planning are effective only when embedded in personalized pathways supported by adequate community resources [9,13]. In this context, it has been demonstrated that continuity of care and regular post-discharge contacts significantly reduce the risk of readmission in patients with heart failure, highlighting the importance of nurse-led follow-up [18]. Furthermore, the literature has emphasized that the quality and safety of home care are essential conditions for the success of hospital-to-community transition pathways [45,46]. Finally, attention is drawn to the need to reorganize community services and invest in the nursing profession to make it more attractive and capable of addressing the growing complexity of home care, in line with the strategies outlined in national and European policies [47,48]. The findings are also consistent with the national regulatory and organizational framework. Ministerial Decree 77/2022 highlights the role of family and community nurses and integrated hospital-to-community models as key tools for reducing readmissions and improving quality of care [29], and recent evidence confirms the relevance of these organizational strategies in the management of chronic conditions [30]. At the European level, fragmentation of long-term care services has been recognized as a critical issue, and the need to develop more inclusive and sustainable models has been emphasized [49]. In this context, the evidence generated contributes to supporting policy strategies aimed at strengthening continuity of care and enhancing the role of the nurse case manager as a central figure in transitional care processes.

### 4.1. Limitations

This study has several limitations that should be considered when interpreting the results. First, the retrospective observational design does not allow causal relationships to be established and only supports the identification of associations. Because the activation of interventions depended on clinical judgment and organizational pathways, selection bias cannot be excluded.

Second, the sample, although including all consecutive eligible cases over a one-year period, was relatively small and derived from a single center, which may limit the generalizability of the findings to settings where structured nurse case management or integrated hospital-to-community care pathways are not implemented. No formal sample size calculation was performed, and the inclusion of multiple predictors in the multivariable models may have increased the risk of overfitting.

Another limitation concerns the unbalanced distribution of exposure groups (e.g., 98 non-readmitted vs. 6 readmitted patients among those receiving combined interventions), which may influence the magnitude of estimated odds ratios in logistic regression models. Therefore, the results should be interpreted cautiously as associations observed within this specific organizational pathway rather than causal effects.

In addition, the analysis relied exclusively on data available in the hospital’s electronic medical records and could not capture readmissions occurring in other hospitals. Important clinical variables such as discharge diagnosis, comorbidity burden, polypharmacy, cognitive status, or socioeconomic factors were not available, and discharge ward was used as a proxy for case mix. Consequently, residual confounding cannot be excluded.

Finally, the relatively limited number of outcome events (65 readmissions) may have contributed to statistical instability in the regression analyses. Moreover, the composite “combined interventions” variable may be analytically coarse, as it groups partially exposed patients with those receiving none and does not allow evaluation of dose–response or specific intervention patterns.

### 4.2. Practical Implications

The findings of this study provide relevant insights for clinical practice and service organization. In particular, the central role of the nurse case manager clearly emerges as a key figure in coordinating protected discharge interventions and ensuring continuity of care. The observed effectiveness of combined interventions confirms the importance of adopting multidimensional approaches that integrate clinical-functional assessment, caregiver support, telephone follow-up, and timely activation of community services. These results highlight the need to invest in organizational models that promote shared, multidisciplinary care and strengthen home healthcare services. Furthermore, the findings support the systematic inclusion of caregiver assessment and early discharge planning within clinical care pathways. For nursing practice, this implies enhancing advanced skills in case management, communication with families, and the use of telehealth tools to anticipate patient needs and reduce inappropriate readmissions. In line with the organizational framework introduced by Ministerial Decree 77/2022 [29], which promotes the development of integrated hospital–community care pathways and the strengthening of territorial services, the organizational model evaluated in this study can also be interpreted through a SWOT analysis highlighting its main strengths, limitations, and potential future developments (Table 5).

## 5. Conclusions

This study found that an integrated protected discharge model coordinated by a nurse case manager and including early activation of home care (ADI), post-discharge telephone follow-up, and social worker involvement was strongly associated with lower odds of 30-day hospital readmission among frail older patients. These findings suggest that the potential benefit of transitional care may lie not in single isolated interventions, but in the multidimensional integration of clinical, functional, and social support strategies, aimed at improving continuity between hospital and community care. However, given the retrospective observational design of this study and the possibility of selection bias and confounding by indication, the results should be interpreted with caution and should not be considered causal evidence of effectiveness. Within these limits, the findings are consistent with current national and European healthcare policies that promote integrated hospital–community care models and emphasize the coordinating role of the nurse case manager in transitional care pathways. Further prospective multicenter studies are needed to confirm these associations and to identify which organizational components of protected discharge models most effectively improve patient outcomes, including quality of life, caregiver experience, and healthcare utilization. Overall, the results suggest that coordinated and multidisciplinary transitional care models may represent a promising organizational strategy to support continuity of care and reduce potentially avoidable readmissions among frail older patients.

## Figures and Tables

**Table 1:** Baseline characteristics according to 30-day readmission status.

Variable	Total (*N* = 200)	No RMI (*N* = 135)	RMI (*N* = 65)	*p*-Value
**Age, median (IQR)**	85 (79–89)	85 (79–90)	84 (78–88)	0.867
**Female, *n* (%)**	127 (63.5%)	84 (62.2%)	43 (66.1%)	0.589
**BRASS, median (IQR)**	20 (16–22)	19 (16–22)	21 (18–23)	0.006
**ADL, median (IQR)**	1 (0–2)	1 (0–2)	1 (0–2)	0.149
**IADL, median (IQR)**	1 (0–2)	1 (0–2)	1 (0–2)	0.508
**Nurse Case Manager, *n* (%)**	105 (52.5%)	99 (73.3%)	6 (9.2%)	<0.001
**Social Worker, *n* (%)**	105 (52.5%)	99 (73.3%)	6 (9.2%)	<0.001
**Telephone follow-up, *n* (%)**	105 (52.5%)	99 (73.3%)	6 (9.2%)	<0.001
**ADI within 24 h, *n* (%)**	164 (82.0%)	114 (84.4%)	50 (76.9%)	0.195
**Combined interventions, *n* (%)**	104 (52.0%)	98 (72.6%)	6 (9.2%)	<0.001

**Table 2:** Bivariate analysis of categorical variables according to 30-day readmission status.

Variable	χ^2^ (df)	*p*-Value
**Sex**	0.293 (1)	0.589
**Nurse case manager**	72.296 (1)	<0.001
**Telephone follow-up**	72.296 (1)	<0.001
**Social worker**	66.836 (1)	<0.001
**ADI within 24 h**	1.682 (1)	0.195
**Combined interventions**	70.571 (1)	<0.001

**Table 3:** Multivariate logistic regression (Model 1): clinical variables and individual interventions associated with 30-day hospital readmission.

Variable	OR	95% CI	*p*-Value
**Nurse case manager**	0.023	0.008–0.064	<0.001
**ADI within 24 h**	3.475	1.384–8.725	0.008
**Age**	0.961	0.907–1.019	0.150
**ADL**	0.757	0.458–1.252	0.303
**IADL**	1.183	0.713–1.964	0.512
**BRASS**	1.192	0.925–1.535	0.192
**Sex**	1.168	0.507–2.692	0.717

Note: ADI = Integrated Home Care. Nurse case manager indicates the involvement of the nurse case manager in the discharge process.

**Table 4:** Multivariate logistic regression (Model 2): predictors of 30-day hospital readmission.

Variable	OR	95% CI	*p*-Value
**Combined interventions**	26.1	10.1–67.5	<0.001
**Age**	0.956	0.906–1.010	0.106
**ADL**	0.820	0.497–1.351	0.436
**IADL**	1.102	0.714–1.703	0.660
**BRASS**	1.086	0.984–1.199	0.100
**Sex**	1.305	0.583–2.922	0.518

Note: Combined interventions were coded as 1 = presence and 0 = absence. Effect estimates should be interpreted with caution because of the unbalanced distribution of exposure groups.

**Table 5:** SWOT analysis of the protected discharge model in relation to Ministerial Decree 77/2022.

Dimension	Elements Emerging from the Model Evaluated in This Study
**Strengths**	Strong coordination ensured by the nurse case manager; multidimensional assessment of frail patients; integration of multiple transitional care interventions (ADI activation, telephone follow-up, social support); improved hospital–community continuity of care.
**Weaknesses**	Observational single-center experience; potential dependence on the availability and organization of territorial services; limited data on additional community-based interventions not systematically recorded in institutional databases.
**Opportunities**	Alignment with the territorial healthcare reorganization promoted by Ministerial Decree 77/2022; expansion of integrated care pathways; strengthening of the role of family and community nurses; development of telemedicine and proactive follow-up strategies.
**Threats**	Workforce shortages in community healthcare services; regional heterogeneity in the implementation of territorial care models; potential fragmentation between hospital and territorial services if coordination mechanisms are not fully implemented.

## Data Availability

The datasets generated and analyzed during the current study are not publicly available due to institutional data protection policies, but anonymized data may be made available from the corresponding authors upon reasonable request.

## Supplementary Materials

The following supporting information can be downloaded at: https://www.mdpi.com/article/10.3390/medicina62030602/s1, STROBE Statement—Checklist of items that should be included in reports of cohort studies.

## References

[1] Foster C.C., Jacob-Files E., Arthur K.C., Hillman S.A., Edwards T.C., Mangione-Smith R. (2017). Provider perspectives of high-quality pediatric hospital-To-home transitions for children and youth with chronic disease. Hosp. Pediatr..

[2] Leppin A.L., Gionfriddo M.R., Kessler M., Brito J.P., Mair F.S., Gallacher K., Wang Z., Erwin P.J., Sylvester T., Boehmer K. (2014). Preventing 30-day hospital readmissions: A systematic review and meta-analysis of randomized trials. JAMA Intern. Med..

[3] Hicks C.W., Canner J.K., Karagozlu H., Mathioudakis N., Sherman R.L., Black J.H., Abularrage C.J. (2019). Contribution of 30-day readmissions to the increasing costs of care for the diabetic foot. J. Vasc. Surg..

[4] Goel A.N., Raghavan G., St John M.A., Long J.L. (2019). Risk Factors, Causes, and Costs of Hospital Readmission After Head and Neck Cancer Surgery Reconstruction. JAMA Facial Plast. Surg..

[5] Dhaliwal J.S., Dang A.K. (2025). Reducing Hospital Readmissions. StatPearls.

[6] Murray F., Allen M., Clark C.M., Daly C.J., Jacobs D.M. (2021). Socio-demographic and -economic factors associated with 30-day readmission for conditions targeted by the hospital readmissions reduction program: A population-based study. BMC Public Health.

[7] Zumbrunn A., Bachmann N., Bayer-Oglesby L., Joerg R. (2022). Social disparities in unplanned 30-day readmission rates after hospital discharge in patients with chronic health conditions: A retrospective cohort study using patient level hospital administrative data linked to the population census in Switzerland. PLoS ONE.

[8] Coffey A., Leahy-Warren P., Savage E., Hegarty J., Cornally N., Day M.R., Sahm L., O’Connor K., O’Doherty J., Liew A. (2019). Interventions to Promote Early Discharge and Avoid Inappropriate Hospital (Re)Admission: A Systematic Review. Int. J. Environ. Res. Public. Health.

[9] Gonçalves-Bradley D.C., Lannin N.A., Clemson L., Cameron I.D., Shepperd S. (2022). Discharge planning from hospital. Cochrane Database Syst. Rev..

[10] Naylor M.D., Aiken L.H., Kurtzman E.T., Olds D.M., Hirschman K.B. (2011). The care span: The importance of transitional care in achieving health reform. Health Aff. Proj. Hope.

[11] Chiu W.K., Newcomer R. (2007). A systematic review of nurse-assisted case management to improve hospital discharge transition outcomes for the elderly. Prof. Case Manag..

[12] Naylor M.D., Brooten D., Campbell R., Jacobsen B.S., Mezey M.D., Pauly M.V., Schwartz J.S. (1999). Comprehensive discharge planning and home follow-up of hospitalized elders: A randomized clinical trial. JAMA.

[13] Hunt-O’Connor C., Moore Z., Patton D., Nugent L., Avsar P., O’Connor T. (2021). The effect of discharge planning on length of stay and readmission rates of older adults in acute hospitals: A systematic review and meta-analysis of systematic reviews. J. Nurs. Manag..

[14] Goldman L.E., Sarkar U., Kessell E., Guzman D., Schneidermann M., Pierluissi E., Walter B., Vittinghoff E., Critchfield J., Kushel M. (2014). Support from hospital to home for elders: A randomized trial. Ann. Intern. Med..

[15] Facchinetti G., Ianni A., Piredda M., Marchetti A., D’Angelo D., Dhurata I., Matarese M., De Marinis M. (2019). Discharge of older patients with chronic diseases: What nurses do and what they record. An observational study. J. Clin. Nurs..

[16] Fønss Rasmussen L., Grode L.B., Lange J., Barat I., Gregersen M. (2021). Impact of transitional care interventions on hospital readmissions in older medical patients: A systematic review. BMJ Open.

[17] Li J., Wang H., Xie H., Mei G., Cai W., Ye J., Zhang J., Ye G., Zhai H. (2014). Effects of post-discharge nurse-led telephone supportive care for patients with chronic kidney disease undergoing peritoneal dialysis in China: A randomized controlled trial. Perit. Dial. Int. J. Int. Soc. Perit. Dial..

[18] Adib-Hajbaghery M., Maghaminejad F., Abbasi A. (2013). The role of continuous care in reducing readmission for patients with heart failure. J. Caring Sci..

[19] Bahr S.J., Solverson S., Schlidt A., Hack D., Smith J.L., Ryan P. (2014). Integrated literature review of postdischarge telephone calls. West. J. Nurs. Res..

[20] Carter J., Hassan S., Walton A., Yu L., Donelan K., Thorndike A.N. (2021). Effect of Community Health Workers on 30-Day Hospital Readmissions in an Accountable Care Organization Population: A Randomized Clinical Trial. JAMA Netw. Open.

[21] Alvarez R., Ginsburg J., Grabowski J., Post S., Rosenberg W. (2016). The Social Work Role in Reducing 30-Day Readmissions: The Effectiveness of the Bridge Model of Transitional Care. J. Gerontol. Soc. Work.

[22] Linertová R., García-Pérez L., Vázquez-Díaz J.R., Lorenzo-Riera A., Sarría-Santamera A. (2011). Interventions to reduce hospital readmissions in the elderly: In-hospital or home care. A systematic review. J. Eval. Clin. Pract..

[23] Tomlinson J., Cheong V.-L., Fylan B., Silcock J., Smith H., Karban K., Blenkinsopp A. (2020). Successful care transitions for older people: A systematic review and meta-analysis of the effects of interventions that support medication continuity. Age Ageing.

[24] Azzellino G., Aitella E., Passamonti  M., Ginaldi  L., De Martinis  M. (2025). Protected discharge and combined interventions: A viable path to reduce hospital readmissions. Eur. J. Intern. Med..

[25] Blaylock A., Cason C.L. (1992). Discharge planning predicting patients’ needs. J. Gerontol. Nurs..

[26] Fitriana I., Setiati S., Rizal E.W., Istanti R., Rinaldi I., Kojima T., Akishita M., Azwar M.K. (2021). Malnutrition and depression as predictors for 30-day unplanned readmission in older patient: A prospective cohort study to develop 7-point scoring system. BMC Geriatr..

[27] Katz S., Ford A.B., Moskowitz R.W., Jackson B.A., Jaffe M.W. (1963). Studies of illness in the aged. The index of ADL: A standardized measure of biological and psychosocial function. JAMA.

[28] Lawton M.P., Brody E.M. (1969). Assessment of older people: Self-maintaining and instrumental activities of daily living. Gerontologist.

[29] Ministero della Salute Decreto 23 maggio 2022, n. 77 (2022). Regolamento Recante la Definizione di Modelli e Standard per lo Sviluppo Dell’assistenza Territoriale nel Servizio Sanitario Nazionale.

[30] Azzellino G., Vagnarelli P., Passamonti M., Mengoli L., Ginaldi L., De Martinis M. (2025). Integrated Hospital–Territory Organizational Models and the Role of Family and Community Nurses in the Management of Chronic Conditions: A Scoping Review. Medicina.

[31] Baxter S., Johnson M., Chambers D., Sutton A., Goyder E., Booth A. (2018). The effects of integrated care: A systematic review of UK and international evidence. BMC Health Serv. Res..

[32] Azzellino G., Passamonti M., Aitella E., Mengoli L., Vagnarelli P., Ginaldi L., Martinis M.D. (2025). Bridging the Gap in Chronic Disease Management: A Nursing Perspective on the Use of Predictive Tools and Telemedicine in the Hospital–Community Transition. Medicina.

[33] Azzellino G., Aitella E., Ginaldi L., Vagnarelli P., De Martinis M. (2025). Use of Digital and Telemedicine Tools for Postoperative Pain Management at Home: A Scoping Review of Health Professionals’ Roles and Clinical Outcomes. J. Clin. Med..

[34] Regulation—2016/679—EN—Gdpr—EUR-Lex. https://eur-lex.europa.eu/eli/reg/2016/679/oj/eng.

[35] von Elm E., Altman D.G., Egger M., Pocock S.J., Gøtzsche P.C., Vandenbroucke J.P. (2008). The Strengthening the Reporting of Observational Studies in Epidemiology (STROBE) statement: Guidelines for reporting observational studies. J. Clin. Epidemiol..

[36] Fitzgerald J.F., Smith D.M., Martin D.K., Freedman J.A., Katz B.P. (1994). A case manager intervention to reduce readmissions. Arch. Intern. Med..

[37] Gilbert T., Occelli P., Rabilloud M., Poupon-Bourdy S., Riche B., Touzet S., Bonnefoy M., PROUST Study Group (2021). A Nurse-Led Bridging Program to Reduce 30-Day Readmissions of Older Patients Discharged from Acute Care Units. J. Am. Med. Dir. Assoc..

[38] Einstadter D., Cebul R.D., Franta P.R. (1996). Effect of a nurse case manager on postdischarge follow-up. J. Gen. Intern. Med..

[39] Sakashita C., Endo E., Ota E., Oku H. (2025). Effectiveness of nurse-led transitional care interventions for adult patients discharged from acute care hospitals: A systematic review and meta-analysis. BMC Nurs..

[40] Tyler N., Hodkinson A., Planner C., Angelakis I., Keyworth C., Hall A., Jones P.P., Wright O.G., Keers R., Blakeman T. (2023). Transitional Care Interventions from Hospital to Community to Reduce Health Care Use and Improve Patient Outcomes. JAMA Netw. Open.

[41] Stranges P.M., Marshall V.D., Walker P.C., Hall K.E., Griffith D.K., Remington T. (2015). A multidisciplinary intervention for reducing readmissions among older adults in a patient-centered medical home. Am. J. Manag. Care.

[42] Landi F., Onder G., Russo A., Tabaccanti S., Rollo R., Federici S., Tua E., Cesari M., Bernabei R. (2001). A new model of integrated home care for the elderly: Impact on hospital use. J. Clin. Epidemiol..

[43] Rodakowski J., Rocco P.B., Ortiz M., Folb B., Schulz R., Morton S.C., Leathers S.C., Hu L., James A.E. (2017). Caregiver Integration during Discharge Planning of Older Adults to Reduce Resource Utilization: A Systematic Review and Meta-Analysis of Randomized Controlled Trials. J. Am. Geriatr. Soc..

[44] Zarovska A., Evangelista A., Boccia T., Ciccone G., Coggiola D., Scarmozzino A., Corsi D. (2018). Development and validation of a simplified BRASS index to screen hospital patients needing personalized discharge planning. J. Gen. Intern. Med..

[45] Bagnasco A., Alvaro R., Lancia L., Manara D.F., Zega M., Rocco G., Rasero L., Mazzoleni B., Sasso L. (2023). Protocol for evaluating quality and safety for the public through home care nursing in Italy: A multicentre cross-sectional descriptive observational study (AIDOMUS-IT). BMJ Open.

[46] Barber B., Gregg E., Macdonald M., Moody E., Rothfus M., Weeks L.E. (2023). Transitional care programs in Canada for older adults transitioning from hospital to home: Protocol for a systematic review of text and opinion. JBI Evid. Synth..

[47] Azzellino G., De Martinis M. (2024). Territorial reorganization, telemedicine and operative centres: Challenges and opportunities for the nursing profession. J. Clin. Nurs..

[48] Azzellino G., Ginaldi L., De Martinis M. (2024). Renew the Nursing Profession to Attract New Forces and Be Increasingly Inclusive and Attentive to Diversity. J. Adv. Nurs..

[49] Spasova S., Baeten R., Coster S., Ghailani D., Peña-Casas R., Vanhercke B. (2018). Challenges in Long-Term Care in Europe: A Study of National Policies.

