# Supplementary material for: Impact of Combined Interventions and Early Home Care Activation on 30-Day Hospital Readmissions: A Retrospective Observational Study
Source: Medicina (Kaunas). 2026 Mar 23;62(3):602. doi: 10.3390/medicina62030602 (PMC13028036; doi:10.3390/medicina62030602)
Supplement: Supplementary file 1 [file medicina-62-00602-s001.zip › medicina-4187802-supplementary.pdf]

STROBE Statement—Checklist of items that should be included in reports of ***cohort studies***

	Item No	Recommendation
**Title and abstract**	1	Page 1
		Page 1
**Introduction**		
Background/rationale	2	Page 2
Objectives	3	Page 2
**Methods**		
Study design	4	Page 3
Setting	5	Page 3
Participants	6	Page 4
		Not applicable
Variables	7	Page 4-5
Data sources/ measurement	8*	Page 4-5
Bias	9	Page 5 and Page 10-11
Study size	10	Page 4
Quantitative variables	11	Page 5
Statistical methods	12	Page 5
		Not applicable
		Page 4-5
		Not applicable
		Not performed
**Results**		
Participants	13*	Page 4
		Not applicable.
		Not applicable (retrospective cohort without staged recruitment)
Descriptive data	14*	(a) Give characteristics of study participants (eg demographic, clinical, social) and information on exposures and potential confounders
		(b) Indicate number of participants with missing data for each variable of interest
		(c) Summarise follow-up time (eg, average and total amount)
Outcome data	15*	Report numbers of outcome events or summary measures over time
Main results	16	Page 6-9
		Not applicable
		Not applicable
Other analyses	17	Not performed
**Discussion**		
Key results	18	Page 9
Limitations	19	Page 10-11
Interpretation	20	Page 9-10
Generalisability	21	Page 9-11
**Other information**		
Funding	22	No external funding. Page 12

*Give information separately for exposed and unexposed groups.

Note: An Explanation and Elaboration article discusses each checklist item and gives methodological background and published examples of transparent reporting. The STROBE checklist is best used in conjunction with this article (freely available on the Web sites of PLoS Medicine at http://www.plosmedicine.org/, Annals of Internal Medicine at http://www.annals.org/, and Epidemiology at http://www.epidem.com/). Information on the STROBE Initiative is available at http://www.strobe-statement.org.
